# Quantifying relative virulence: when *μ*_max_ fails and AUC alone just is not enough

**DOI:** 10.1099/jgv.0.001515

**Published:** 2020-11-05

**Authors:** Ruben Michael Ceballos, Carson Len Stacy

**Affiliations:** ^1^​ Department of Biological Sciences, The University of Arkansas, Fayetteville, AR, USA; ^2^​ Arkansas Center for Space and Planetary Sciences, Fayetteville, AR, USA; ^3^​ Cell and Molecular Biology Program, The University of Arkansas, Fayetteville, AR, USA

**Keywords:** virus–host dynamicsx, growth curve analysis, relative virulence, non-lytic viruses, Sulfolobus spindle-shaped virus, Gompertz model

## Abstract

A challenge in virology is quantifying relative virulence (*V*_R_) between two (or more) viruses that exhibit different replication dynamics in a given susceptible host. Host *growth curve analysis* is often used to mathematically characterize virus–host interactions and to quantify the magnitude of detriment to host due to viral infection. Quantifying *V*_R_ using canonical parameters, like maximum specific growth rate (*μ*_max_), can fail to provide reliable information regarding virulence. Although area-under-the-curve (AUC) calculations are more robust, they are sensitive to limit selection. Using empirical data from Sulfolobus Spindle-shaped Virus (SSV) infections, we introduce a novel, simple metric that has proven to be more robust than existing methods for assessing *V*_R_. This metric (*I*_SC_) accurately aligns biological phenomena with quantified metrics to determine *V*_R_. It also addresses a gap in virology by permitting comparisons between different non-lytic virus infections or non-lytic versus lytic virus infections on a given host in single-virus/single-host infections.

## Full-Text

Two of the more difficult aspects of quantitative virology are accurate determination of virus titre, and comparing relative virulence between two (or more) viruses on a given host when virus–host infection dynamics are distinct for each virus. For the former, the community has settled on several methods for quantifying virus ‘titre’ (an essential for calculating multiplicity of infection; MOI). These include serial dilution plate-based plaque assays, qPCR-based titres, TEM-based virometry, ESI/MS, and, more recently, flow virometry. Each method has noted shortcomings. Some methods overestimate (e.g. qPCR, ESI/MS) while others underestimate (e.g. plaque assays) the actual number of infectious virions per unit volume [1–4]. Several of these same methods are used to address the latter question of *relative virulence* (*V*_R_) between two (or more) strains of virus separately infecting the same host (or the same host species). In reality, most of these metrics simply provide a measure of virus production rate or virus count, which is then correlated to transmission rate. However, transmission rate does not always provide accurate information about relative virulence. Even low-virulence persistent viral infections can be highly productive in terms of virion yield or have high transmission rates (e.g. herpesviruses). Two other metrics – namely, ID_50_ and LD_50_ – have utility for quantifying highly pathogenic and virulent infections (e.g. ebolaviruses). ID_50_ is the infectious dose required to cause infection in 50 % of the affected host population. In tissue culture (i.e., *in vitro*), this is referred to as TCID_50_. LD_50_ is the lethal dose at which 50 % of the affected host population perishes due to the infection. Although ID_50_ and LD_50_ are useful for some *in vivo* and *in vitro* models (and in epidemiology), these and the other aforementioned metrics have limitations when attempting to determine *V*_R_. Determining *V*_R_ is particularly challenging when two (or more) viruses under study exhibit different replication dynamics on a given host. Therefore, *host growth curve analysis* is commonly used to elucidate details of virus–host dynamics and determine relative virulence.

Although host growth curve analysis is standard practice in experimental infections to characterize virus–host interactions and to mathematically calculate the detriment a virus levies on host growth, assessing *V*_R_ using canonical measures of fitness, such as maximum specific growth rate (*µ*_max_) [5], can fail to accurately describe experimental infection data [6], especially for non-lytic viruses. In non-lytic virus systems, progeny virions are released via *budding* rather than gross cell lysis and growth curves for hosts infected with non-lytic viruses can exhibit non-canonical growth profiles. For example, since the experimental infection is typically initiated once a cell culture is viable (i.e. at a defined cell density and typically in early- or mid-exponential phase growth), the resulting host growth curve during infection will lack a *lag* phase and may feature brief *exponential* growth and a prolonged period of non-exponential (but positive) growth prior to reaching *stationary* phase.

Using empirical data from SSSV infections, we introduce a novel, yet simple metric that overcomes limitations of traditional growth curve analysis when quantifying relative virulence between two viruses independently infecting a common host at a constant starting MOI. This approach (viz: Stacy–Ceballos equations; see equations 4, 5 and 6) more accurately aligns biological phenomena with quantified metrics for *V*_R_ and addresses a gap in virology by allowing comparisons between non-lytic (or non-lytic versus lytic) infections. In this study, we demonstrate that the relative decrease in *maximum specific growth rate* (*µ*_max_) and percent inhibition based on area-under-the-curve (i.e. PI_AUC_) between uninfected and infected liquid cultures of susceptible host are inadequate for reliably determining *V*_R_ between different strains of SSV in single-virus/single-host (SVSH) infections.

**Fig. 1.**
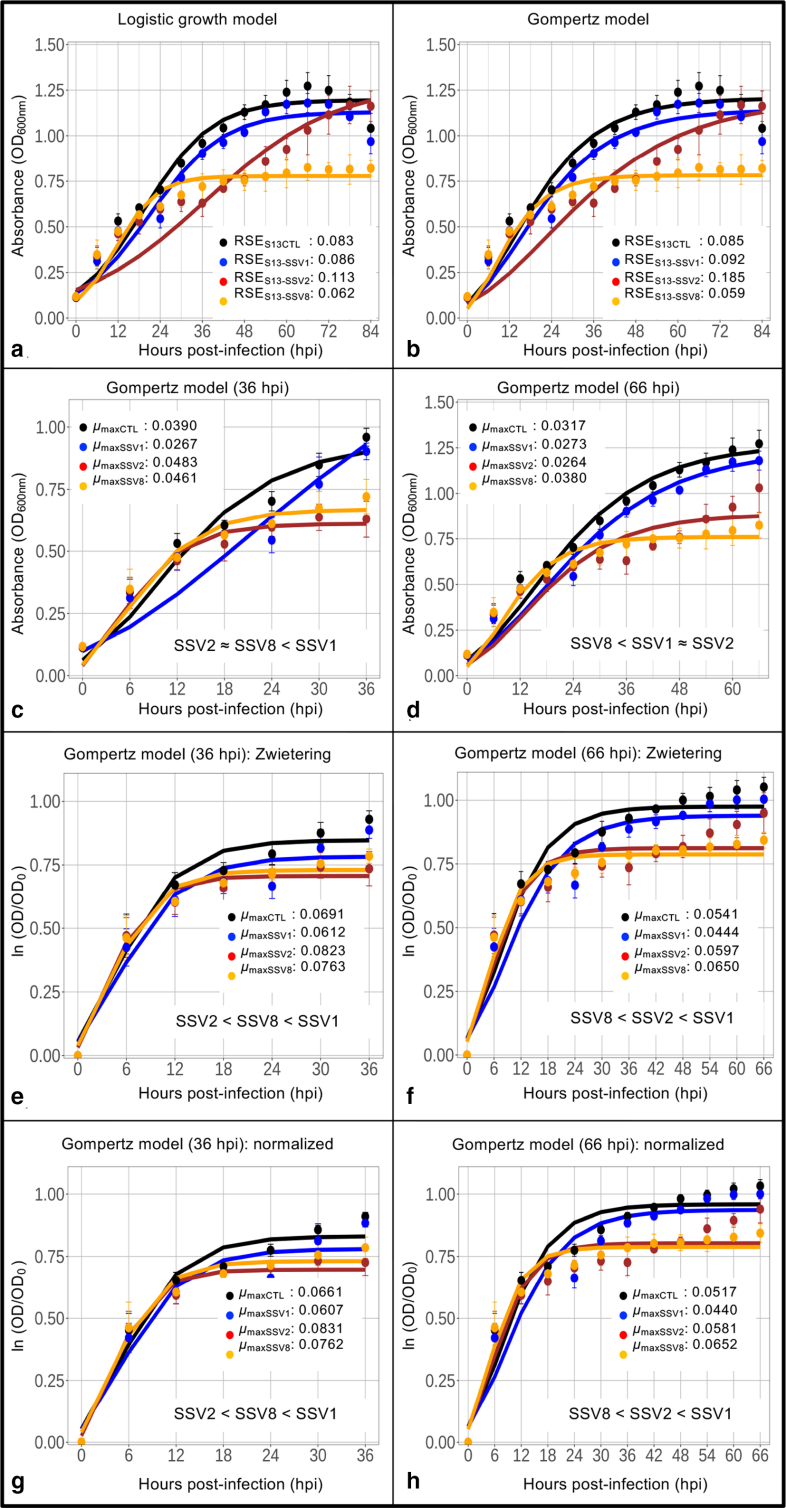
Growth curve analysis for SSV data using maximum specific growth rate (*μ*_max_). Growth curves were generated using host *Sulfolobus* strain Gθ [7, 10] infected with SSV1 [26], SSV2 [12] and SSV8 [27], in single-host/single-virus trials at a MOI=0.1 at 78 °C and pH 3.2. Growth curves are shown for uninfected host control (black), SSV1-infected strain Gθ (blue), SSV2-infected strain Gθ (red) and SSV8-infected strain Gθ (orange). (a) Logistic growth model fit to the raw host growth curve data with sd values. (b) Gompertz model fit to the raw host strain Gθ growth data with sd values. (c) Maximum specific growth rate (*μ*_max_) of the Gompertz model fit over a narrower range of 0–36 hpi representing classical log phase. (d) Expanded growth interval to stationary phase (0–66 hpi) with *μ*_max_ values for the Gompertz fit. (e) Gompertz of log-transformed data [14] with *μ*_max_ values for the truncated dataset (0–36 hpi). (f) Gompertz of log-transformed and normalized data with *μ*_max_ values for the expanded host growth curve data (0–66 hpi). (g) Gompertz of log-transformed data with normalization for start point cell density (0–36 hpi). (h) Gompertz of log-transformed data with normalization for start point cell density (0–66 hpi). Based on *μ*_max_ values for the Gompertz non-log-transformed and log-transformed model fits, the order of relative virulence for the viruses (SSV1, SSV2, SSV8) under comparison is provided with least virulent to the left and most virulent to the right. There is no agreement between the analytical treatments even when data are truncated or expanded.

SSVs are non-lytic double-stranded DNA viruses that infect species of the family *Sulfolobaceae* – a group of hyperthermophilic archaea. *V*_R_ across three SSVs was assessed by comparing parameters between growth curves from host cultures, each of which was infected with one of three viruses: SSV1 [7], SSV2 [8] or SSV8 [9] – in SVSH infections on the host, *Sulfolobus* strain Gθ [7, 10]. Absorbance data (a proxy for cell density) were fit with modified Logistic and Gompertz models. Both model types exhibit similar *goodness of fit* (Fig. 1a, b); however, Gompertz models [8] are preferred for analysing diseased cells [9, 11].

Since, SSVs do not form true plaques on host lawns but rather diffuse turbid halos [7, 11, 12], MOI was determined using ‘halo assays’. These halo assays are serial dilution plaque-like plate assays with units of halo-forming units per millilitre (hfu ml^−1^) [11], similar to the plaque-forming units per millilitre (pfu ml^−1^) or infectious units per millilitre (ifu ml^−1^) commonly used to define titre in bacteriophage and other virus systems amenable to growing homogenous host lawns on plates.

*In comparing host growth using maximum growth rate* (*µ*_max_ *) as a metric for relative virulence,* two different intervals were considered. First, an interval from 0 to 36 hours post-infection (hpi), which best represents the archetypal ‘exponential growth phase’ [13] was considered (Fig. 1c). Using *µ*_max_ from the Gompertz, SSV2 and SSV8 show similar high maximum specific growth rates indicating low virulence while SSV1 appears to be the most virulent (Fig. 1c). Given that host growth subject to non-lytic viral infection does not always exhibit a classical Monodian profile, an outer bound at 66 hpi was used to capture more of the growth curve (Fig. 1d). Calculating *µ*_max_ from the Gompertz for this larger portion of the data changes the results. Specifically, SSV8 appears to be the least virulent, while SSV1 and SSV2 exhibit an approximately equal virulence according to *µ*_max_ estimates (Fig. 1d). Thus, for non-lytic infections, a significant change in *µ*_max_, which drives interpretation of results, can emerge depending on how much of the curve is considered. Depending on culture size and specific virus–host pairing, the truly *exponential* growth phase may be brief with the majority of positive growth comprising the classically described *deceleration*, before *stationary* phase.

A widely used and agreed upon alternative is to calculate *µ*_max_ from a log-transformed dataset [14]. Calculating *µ*_max_ from log-transformed data (i.e. ln OD/OD_0_) using narrow (0–36 hpi) and expanded (0–66 hpi) intervals yields another outcome. Comparing early growth, SSV2 appears to be least virulent followed by SSV8 while SSV1 has the lowest *µ*_max_ (Fig. 1e). The expanded interval of the log-transformed data suggests SSV8 is the least virulent followed closely by SSV2 while SSV1 emerges as the most virulent (Fig. 1f). Adding an additional normalization step to compensate for different host cell density measurements at time of viral inoculation (*t*_0_), yields slightly different estimations, but with the same trends as log-transformed data (Fig. 1g, h). Remarkably, none of these analytical adjustments for *µ*_max_, the principal parameter for relative virulence, captures the known relationship of SSV1, SSV2, and SSV8 virulence on *Sulfolobus* strain Gθ [7, 11]. Thus, methods for determining *V*_R_, using *µ*_max_ as a key parameter, are inadequate.

*In comparing host growth using AUC as a metric for relative virulence*, two sets of limits were also used. Given the demonstrated inadequacy of *µ*_max_ in determining *V*_R_ in non-lytic viral infections, an alternative approach is to calculate a percent inhibition (PI_AUC_) of host growth [15–18] based on AUC for infected (AUC_infected_) and uninfected controls (AUC_CTL_).

(1) $$AUC=\sum_{i=0}^{n-1}\frac{1}{2}\left(OD_{i}+OD_{i+1})\cdot (t_{i}+t_{i+1}\right)$$

yields PI_AUC_ on non-log-transformed cell density data, given by

(2) $$PI_{AUC}=\frac{\left({AUC}_{CTL}-{AUC}_{infected}\right)}{{AUC}_{CTL}}\cdot 100.$$

This may be alternatively written as:

(3) $$PI_{AUC}=(1-\frac{AUC_{infected}}{AUC_{CTL}})\cdot 100.$$

When examining *V*_R_ based on PI_AUC_, selection of upper and lower bounds of integration are critical [19]. Yet, approaches for choosing these bounds vary between studies and are often arbitrary [16, 17]. For comparing the phenotypic effects of viral infection, the time of inoculation (*t*_0_) is a reasonable lower bound so that early changes in host growth may be captured. In many reports, the time point corresponding to the upper bound of integration is selected absent of any noted mathematical or biological explanation. Historically, the selection of bounds has been subjective. Prior work (e.g. in cancer biology) has relied on predefined *end-points* after culture initiation [20, 21]. It is generally agreed that a reasonable upper bound is the beginning of *stationary* phase or *peak growth* (i.e. *N*_asymptote_). However, non-canonical host growth during infection may render this value difficult to determine.

**Fig. 2.**
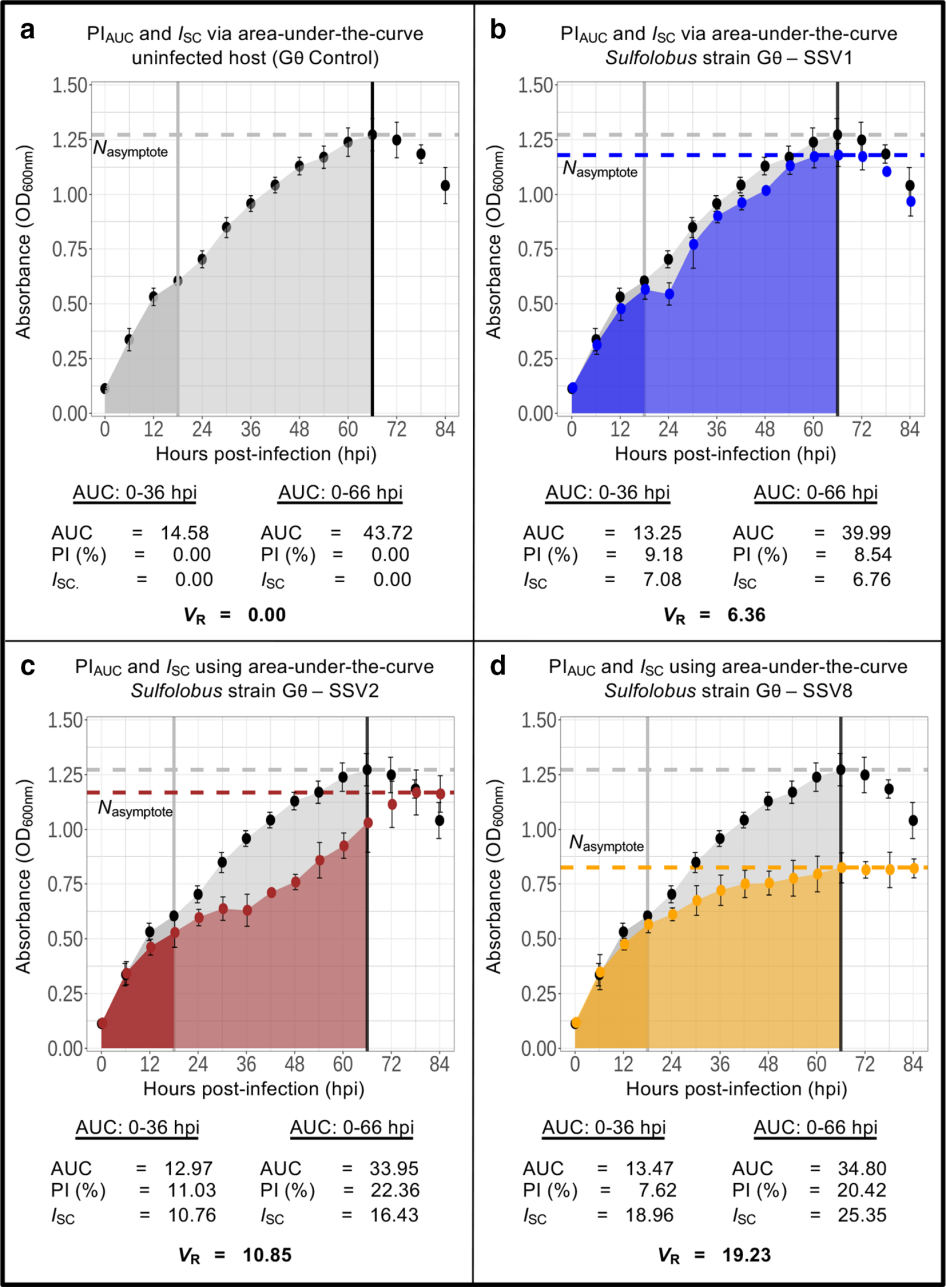
Growth curve analysis of SSV data: AUC. Growth curves for archaeal host *Sulfolobus* strain Gθ [7, 10] infected with SSV1 [26], SSV2 [12] and SSV8 [27], in single-host/single-virus trials at MOI=0.1 (78 °C, pH 3.2). AUC for: (a) uninfected control (black); (b) SSV1-infected host *Sulfolobus* strain Gθ (blue); (c) SSV2-infected *Sulfolobus* strain Gθ (maroon); and (d) SSV8-infected host *Sulfolobus* strain Gθ (gold). AUC, PI_AUC_, and *I*_SC_ were calculated for each SSV-infected host growth curve (and uninfected control) using two different sets of integration bounds: 0-36 hpi and 0-66 hpi. Peak growth (i.e. carrying capacity) is denoted by *N*_asymptote_ for the uninfected control and each of the infected host cultures. Error bars represent the sd from the average of three independent measurements.

**Fig. 3.**
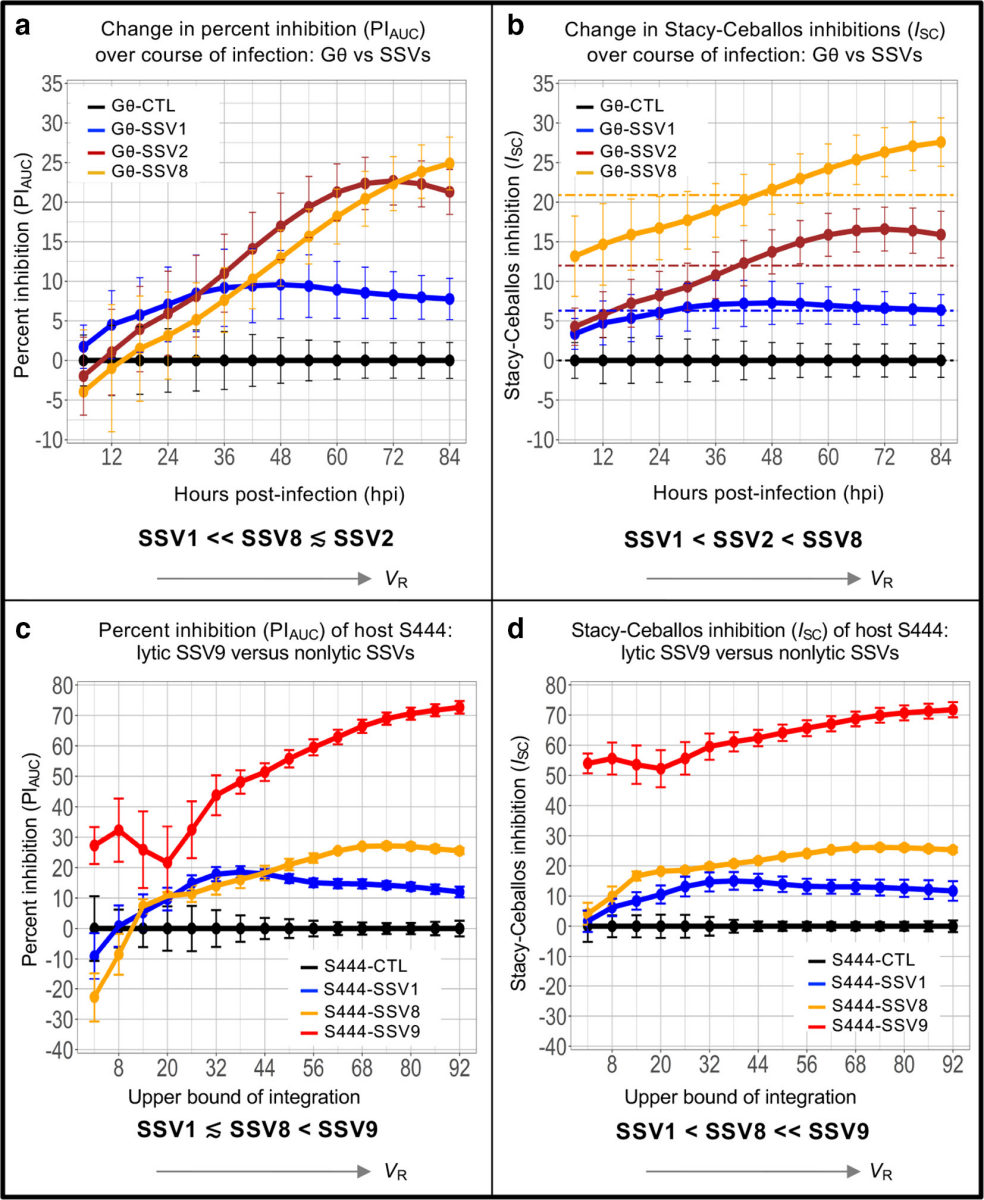
Changes in PI_AUC_ and *I*_SC_ values based on chosen domain of integration. Each point represents the calculated PI_AUC_ for the growth curve from *t*_0_ to each measured time point. (a) PI_AUC_ as a function of the selected upper bound of integration based on *Sulfolobus* strain Gθ host growth curves (shown in Fig. 2); (b) Stacy–Ceballos Inhibition (*I*_SC_) as a function of the selected upper bound of integration for the Gθ dataset; (c) PI_AUC_ as a function of the selected upper bound of integration for *Sulfolobus* sp. strain S444 [7] infected with non-lytic SSV1 (blue) [3], SSV8 (gold) [27] and lytic-type strain SSV9 (red) [7, 10, 27] in single-host/single-virus trials at MOI=0.1 (78 °C, pH 3.2); (d) The calculated inhibition of growth (*I*_SC_) as a function of the selected upper bound of integration for the *Sulfolobus* sp. strain S444 dataset. Error bars represent the sd from the average of three independent measurements.

Using extremes for the outer limit at 36 hpi and 66 hpi for the *Sulfolobus* strain Gθ-SSV dataset (Fig. 2), AUC is calculated. Given that truly ‘exponential’ growth can be brief for non-lytic infections (and even for uninfected controls) followed by a long non-exponential growth phase, 36 hpi represents a conservative upper bound. Alternatively, the upper bound at 66 hpi incorporates more of the data, extending deeply into the positive non-exponential growth phase and capturing the growth peak of the uninfected control curve (Fig. 2). Bound at 36 hpi, some AUC calculations indicate that SSV8 ≲ SSV1 ≲ SSV2, which is not the correct relative virulence between these viruses. Comparing the AUC of the first 36 h of growth does not accurately represent relative virulence across all virus–host comparisons. For example, in *Sulfolobus* sp. Strain S444 (Fig. 3c), SSV8 is known to be more virulent than SSV1, despite similar AUC values at 36 hpi. Moreover, less than half of the dataset is represented, rendering results unconvincing.

To capture a larger component of the virus–host interaction through the peak growth (*N*_asymptote_) of the uninfected control data, the bound was moved to 66 hpi, yielding: SSV1 <<SSV8 ≲ SSV2. This is inaccurate and demonstrates that assessing virulence using PI_AUC_ is unreliable and sensitive to limit selection. What is needed is a reliable metric that captures a significant component of the virus–host interaction (i.e. to peak growth) while also yielding the correct *V*_R_ between viruses.

*In comparing host growth using the Stacy–Ceballos index, a novel measure for relative virulence* is derived. Although *µ*_max_ and AUC are useful parameters for characterizing drug interactions [22] or attenuated/enhanced growth in mutant versus wild-type cell growth [16, 23, 24], when comparing virulence between non-lytic viruses on a host, values for these parameters will depend on integral limit selection. A key component of virus–host interactions is *N*_asymptote_ (i.e. peak host growth), which is a critical but often ignored parameter in growth curve analysis [19]. By considering both percent inhibition of the growth phase as well as the percent inhibition in *N*_asymptote_, a more robust representation of *V*_R_ can be determined. Notably, the square root of the product of PI_AUC_ and PI_max_, introduced here as *Stacy–Ceballos* inhibition (*I*_SC_), provides a robust index of *V*_R_, where

(4) $$PI_{max}=(1-\frac{N_{asymptote(infected)}}{N_{asymptote(control)}})\cdot 100.$$

Such that

(5) $$I_{SC}=[PI_{AUC}\cdot PI_{max}{]}^{1/2}.$$

Using *I*_SC_, the correct order of increasing virulence emerges (i.e. SSV1 <SSV2<SSV8) for both 36 hpi and 66 hpi limits (Fig. 3b) with the latter representing a broad range across the virus–host dynamic (Fig. 2a–d). Thus, *I*_SC_ is a robust index that is resilient to differences in limit selection.

Cautions against combining parameters into a single metric are acknowledged [25]; however, *I*_SC_ allows inclusion of relevant differences in growth kinetics of infected hosts at time points after the control group has reached stationary phase. (Note the growth of SSV2-infected host strain Gθ in Fig. 2). Current approaches for calculating AUC would not account for this continued growth.

Using percentage measures permits meaningful comparisons across virus–host systems and different MOI values. Growth curves with similar growth patterns will typically result in an *I*_SC_ similar to PI_AUC_. However, *I*_SC_ provides a more reliable quantification of differences between growth curves that exhibit distinct growth patterns.

A measure of relative virulence (*V*_R_) calculated by taking the mean of the integrand of *I*_SC_ values (as described below in equation 6) results in a simple yet informative value that is not arbitrarily defined by the researcher and ensures early effects on growth are incorporated into a quantified *V*_R_ [19]. Specifically,

(6) $$V_{R}=\sum_{i=1}^{n-1}\frac{\left(I_{Sci}+I_{SCi+1})\cdot (t_{i}-t_{i+1}\right)}{2(t_{n}-t_{1})}$$

such that *n* is the number of observations from time of infection to the time at which the control growth curve reaches *N*_asymptote_ or peak density if *N*_asymptote_ cannot be determined. (These numbers are represented as dotted lines on Fig. 3b, d). The cessation of integration at time *t_n_* avoids repeated measures on the same value of the control growth curve. For the *Sulfolobus* strain Gθ dataset, the *V*_R_ values calculated via this approach are provided at the bottom of each panel of Fig. 2 and are represented as horizontal dashed lines in the right column of Fig. 3. For *Sulfolobus* sp. strain S444, *V*_R_ with 95 % confidence margins are shown for: SSV1=11.59 (±2.11); SSV8=23.60 (±0.97) and SSV9=60.75 (±7.38) – and match expected patterns.

*Stacy–Ceballos Inhibition (I*_SC_ *) as metric for relative virulence is generalizable to other systems* including comparisons between non-lytic and lytic virus infections on the same susceptible host. The non-lytic SSV system, provides one example of how traditional parameters for assessing relative virulence (i.e. *µ*_max_ and AUC) between two (or more) viruses on a given host may yield unreliable results and incorrect interpretations of infection data. Using Stacy–Ceballos inhibition (*I*_SC_) as a metric for calculating relative virulence overcomes the sensitivity of these parameters providing a more robust and reliable approach for determining *V*_R_.

This approach is not constrained to non-lytic viruses. It is also useful when comparing non-lytic versus lytic infections. In this case, PI_max_ for the lytic system would be the maximum cell density achieved prior to lysis. The ability to accurately assess differences in virulence between lytic viruses and non-lytic viruses or changes in virulence as a virus switches between non-lytic (but productive) and lytic phases offers new opportunities in characterizing single-virus/single-host interactions. In a separate report, reliability and robustness of this approach is demonstrated for other applications in microbiology [19]. We are also assessing the applicability of *I*_SC_ in polymicrobial infections, including multi-virus/single-host (MVSH) infections.
